# Supplementary material for: Natural hybridization in heliconiine butterflies: the species boundary as a continuum
Source: BMC Evol Biol. 2007 Feb 23;7:28. doi: 10.1186/1471-2148-7-28 (PMC1821009; doi:10.1186/1471-2148-7-28)
Supplement: Additional File 1 — Hybrids between species of Heliconius and Eueides butterflies: a database. HTML file linking to database of all known wild-caught interspecific hybrid specimens in the Heliconiina, consisting of introductory text, a list of specimens, together with collection data and photographs of the specimens, and links to information about some artificial hybrids and mutants in the group. This is an edited copy of our online database of Heliconius hybrids [102]. To view database, download zip file and extract to a separate folder, then open index.html within that folder. [file 1471-2148-7-28-S1.zip › mavarez/cristobaltab.html]

Collection of Heliconius melpomene, H. cydno and hybrids from Venezuela
**A collection of
*Heliconius cydno*, *H. melpomene* and hybrids from San Cristobal, Táchira, Venezuela**
  
© James Mallet & Jesús Mavárez 2003
  
**Click number at left to see pictures**
  
Back to hybridization document
  
J. Mallet home page
  
**Last updated:** Friday, 9 January 2004  
Note: These collections were made by Jesús Mavárez
in forest forming part of the botanic garden at San Cristobal, Táchira,
Venezuela in October and December 2003.  *Heliconius melpomene* here mimics *H. erato hydara*, but *H. cydno cordula*
has no known mimetic allegiance; the usual co-mimic *H. sapho* is absent from Venezuela, and the other possible co-mimic,
*H. eleuchia*, is very rare (a single individual has been recorded in Venezuela, further North) and is not involved
in mimicry with *H. cydno*.
The populations seem to have undergone hybridization in the past, and
now have backcross phenotypes at relatively high frequency.

|  |  |  |
| --- | --- | --- |
| No | Species or hybrid type | Sex |
| 1 | Heliconius cydno cordula | m |
| 2 | Heliconius cydno cordula | m |
| 3 | Heliconius cydno cordula | m |
| 4 | Heliconius cydno cordula | f |
| 5 | Heliconius cydno cordula | m |
| 6 | Heliconius cydno cordula | m |
| 7 | Heliconius cydno cordula | m |
| 8 | Heliconius cydno cordula | m |
| 9 | Heliconius cydno cordula | m |
| 10 | Heliconius cydno cordula | f |
| 11 | Heliconius cydno cordula | m |
| 12 | Heliconius cydno cordula | m |
| 13 | Heliconius cydno cordula | f |
| 14 | Heliconius cydno cordula | m |
| 15 | Heliconius cydno cordula | m |
| 16 | Heliconius cydno cordula | m |
| 17 | Heliconius cydno cordula | m |
| 18 | Heliconius cydno cordula | m |
| 19 | Heliconius cydno cordula | f |
| 20 | Heliconius cydno cordula | m |
| 21 | Heliconius cydno cordula | m |
| 22 | Heliconius cydno cordula | m |
| 23 | Heliconius cydno cordula | m |
| 24 | Heliconius cydno cordula | m |
| 25 | Heliconius cydno cordula | f |
| 26 | Heliconius cydno cordula | f |
| 27 | Heliconius cydno cordula | m |
| 28 | Heliconius cydno x melpomene hybrid (BC -> cydno) | m |
| 29 | Heliconius cydno x melpomene hybrid (BC -> cydno) | f |
| 30 | Heliconius cydno x melpomene hybrid (BC -> cydno) | m |
| 31 | Heliconius cydno x melpomene hybrid (BC -> cydno) | m |
| 32 | Heliconius cydno x melpomene hybrid (BC -> cydno) | f |
| 33 | Heliconius cydno x melpomene hybrid (BC -> cydno) | m |
| 34 | Heliconius cydno x melpomene hybrid (BC -> melpomene) | m |
| 35 | Heliconius melpomene melpomene | m |
| 36 | Heliconius melpomene melpomene | m |
| 37 | Heliconius melpomene melpomene | m |
| 38 | Heliconius melpomene melpomene | m |
| 39 | Heliconius melpomene melpomene | m |
| 40 | Heliconius melpomene melpomene | m |
| 41 | Heliconius melpomene melpomene | m |
| 42 | Heliconius melpomene melpomene | m |
| 43 | Heliconius melpomene melpomene | m |
| 44 | Heliconius melpomene melpomene | m |
| 45 | Heliconius melpomene melpomene | m |
| 46 | Heliconius melpomene melpomene | m |
| 47 | Heliconius melpomene melpomene | m |
|  | + 33 more Heliconius cydno |  |
|  | + 23 more Heliconius melpomene |  |
